# Supplementary material for: Chemokinesis-driven accumulation of active colloids in low-mobility regions of fuel gradients
Source: Sci Rep. 2021 Feb 26;11:4785. doi: 10.1038/s41598-021-83963-x (PMC7910604; doi:10.1038/s41598-021-83963-x)
Supplement: Supplementary file 3 — Supplementary Information. [file 41598_2021_83963_MOESM3_ESM.docx]

**Chemokinesis-Driven Accumulation of Active Colloids in Low-Mobility Regions of Fuel Gradients**

**Supplementary Information**

Jeffrey L. Moran*^1‡^, Philip M. Wheat^2‡^, Nathan A. Marine^2^, & Jonathan D. Posner^3,4,5^

^1^Department of Mechanical Engineering, George Mason University, Fairfax, VA

^2^Ira A. Fulton Schools of Engineering, Arizona State University, Tempe, AZ

^3^Department of Mechanical Engineering, University of Washington, Seattle, WA

^4^Department of Chemical Engineering, University of Washington, Seattle, WA

^5^Department of Family Medicine, School of Medicine, University of Washington, Seattle, WA

(^‡^ These authors contributed equally)

(* Corresponding author)

**Concentration Profiles of H_2_O_2_ and KCl**

To better understand the observed response of the rods to their immediate environment, we estimate the concentration distributions of hydrogen peroxide (H_2_O_2_) and potassium chloride (KCl) in the main channel in which the chemokinesis assay is performed. Since there is no bulk fluid flow in the main channel and solute advection is therefore negligible, the concentration profiles for both species can be approximated using Fick’s Second Law, which dictates that the time rate of change of concentration at a point is proportional to the spatial gradient in flux there. At steady state, the spatial gradient in flux is zero. Therefore, the total rate of transport (flux integrated over the entire area) of KCl or H_2_O_2_ is constant along *x*,

$$-AD\frac{dC}{dx}=\text{constant,}$$

$$\Rightarrow A_{1}D_{1}\frac{dC}{dx_{1}}=A_{2}D_{2}\frac{dC}{dx_{2}},$$

where *A* is the cross-sectional area through which diffusion occurs, *D* is the mass diffusivity, *C* is species concentration, *x* is the horizontal spatial coordinate, and 1 and 2 indicate two different *x-*locations, “1” being inside the pores of the nitrocellulose membranes and “2” being in the main channel. The diffusivities of both KCl and H_2_O_2_ can be assumed uniform throughout the pores and the channels. (Pores of diameter 0.4 μm are sufficiently large to not be concerned with ion exclusion.) Therefore, for both H_2_O_2_ and KCl,

$$A_{1}\left(\frac{dC}{dx}\right)_{1}=A_{2}\left(\frac{dC}{dx}\right)_{2}.$$

With a membrane porosity of 50%, *A_1_* = *A_2_*/2, where *A_1_* is the cross-sectional area of the membrane and *A_2_* is the cross-sectional area perpendicular to the direction of diffusion in the channel. Therefore,

$$\left(\frac{dC}{dx}\right)_{membrane}=2\left(\frac{dC}{dx}\right)_{center\_channel}.$$

Because the gradients are linear and there are two membrane walls and one center channel, this can be rewritten as:

$$\left(\frac{\Delta C}{\Delta x}\right)_{membrane}=2\left(\frac{\Delta C}{\Delta x}\right)_{center\_channel}.$$

$$\Rightarrow\frac{\Delta C_{membrane}}{2w_{membrane}}=2\frac{\Delta C_{center\_channel}}{w_{center\_channel}}.$$

where

$$\Delta C_{membrane}+\Delta C_{center\_channel}=\Delta C_{total}.$$

The membrane wall thicknesses are 1 mm and the center channel width is 0.4 mm. Therefore, *w_membrane_* = 2.5*w_center_channel_* and

$$\Delta C_{membrane}=10\Delta C_{center\_channel},$$

Using the known values for *w_membrane_* and *w_center_channel_* and combining , , and gives

$$\Delta C_{center\_channel}=\frac{\Delta C_{total}}{11}.$$

The change in concentration across the center channel is 1/11 the total concentration drop between the outer channels. In other words, if 5% (w/w) H_2_O_2_ were introduced to the left channel, the maximum concentration of H_2_O_2_ the rods would encounter on the left side of the channel would be 2.72 wt.% while the minimum concentration would be 2.27 wt.% on the right side, a total change of 0.45 wt.% across the main channel. This would result in a gradient in rod motility that is too shallow to result in observable orthokinesis. To steepen the gradient in motility, we introduce 30 wt.% H_2_O_2_ into the left channel, resulting in a change of 2.7 wt.% H_2_O_2_ across the main channel. Specifically, *C_min,H2O2_* = 13.6 wt.%, and *C_max,H2O2_* = 16.3 wt.%. To further amplify the gradient in rod motility, we introduce salt into the right channel, which tends to slow the motion of Pt/Au rods [1], [2]. KCl was selected as the salt because the diffusivities of K^+^ and Cl^-^ ions are very similar, minimizing the development of a diffusion potential that would amplify the diffusiophoretic response of the rods to the gradient in salt concentration [3]. 100 μM KCl + 1 μM fluorescein was used as the salt solution (fluorescein allows visualization and quantification of the KCl gradient, as discussed in the main text). The minimum and maximum concentrations of KCl and fluorescein are then calculated as *C_min,KCl_* = 45.5 μM, *C_max,KCl_* = 54.5 μM. *C_min,Fl_* = 455 nM, and *C_max,Fl_* = 545 nM.

**Relation between effective diffusivity and self-propelled speed**

To predict the ability of the rods to exhibit global behavior analogous to biological chemokinesis, we model the rods’ motion as an enhanced random walk behavior quantified by an effective diffusivity *D_eff_*. Such a representation is used by Howse et al. [4] to model the behavior of a self-motile Janus sphere, using the following equation:

$$D_{eff}=D+\frac{1}{4}\frac{U^{2}}{D_{rot}},$$

where *D_eff_* is the effective diffusivity, *U* is the translational velocity, *D_rot_* is the rotational diffusivity, and *D* is the diffusivity due to Brownian motion in the absence of a chemical promoting a chemokinetic response. Using slightly different assumptions, some authors give a coefficient of 1/6 on the second term on the right-hand side of , [5, p. 201]; this reference also ignores the Brownian diffusivity in comparison with the second term, often called the “swim diffusivity.” Chemokinetic responses imply that the advective velocity and/or rotational diffusivity are functions of a chemical concentration, i.e. *U* = *f*_1_(*C_fuel/nutrients_*) for orthokinesis and/or *D_rot_* = *f*_2_(*C_fuel/nutrients_*) for klinokinesis. From the above equation for effective diffusivity, a chemokinetic response can be described in terms of the effective diffusivity as a function of chemical concentration, i.e. *D_eff_* = *f*(*C_fuel/nutrients_*). Thus, spatial variations in KCl and/or H_2_O_2_ concentration imply a spatial variation in effective diffusivity. As a result, the flux of chemokinetic objects in a spatial gradient of fuel/nutrient concentration can be expressed using the generalization of Fick’s law that deals with significant spatial variations in diffusivity for a Brownian particle.

**Derivation of Fokker-Planck equation used in PDE model**

In this section we derive the transport equation to be used in the PDE model for comparison with the experiments and Brownian Dynamics simulations. Consider a Brownian particle that has a directional speed *u* and a turning frequency *f*. The rotation of the particle is random such that halfway through turning around, an individual particle is equally likely to complete the direction change as it is to return to the original direction. In this case the average frequency of a direction change is *f*/2. Let *R* be the number density of particles moving right along the one-dimensional (*x*) axis, and *L* be the number density of particles moving left. We can write a particle conservation equation as:

$$\frac{\partial R}{\partial t}=-\frac{\partial uR}{\partial x}+\frac{f}{2}L-\frac{f}{2}R,$$

and

$$\frac{\partial L}{\partial t}=\frac{\partial uL}{\partial x}-\frac{f}{2}L+\frac{f}{2}R.$$

The total number density of particles *ρ* = *R+L*, and the particle flux (*J*) is *u*(*R*–*L*). Adding the conservation equations yields

$$\frac{\partial R}{\partial t}+\frac{\partial L}{\partial t}=-\frac{\partial uR}{\partial x}+\frac{\partial uL}{\partial x}\Rightarrow\frac{\partial\left(R+L\right)}{\partial t}=-\frac{\partial u\left(R-L\right)}{\partial x}\Rightarrow\frac{\partial\rho}{\partial t}=-\frac{\partial J}{\partial x},$$

and subtracting the two equations yields

$$\frac{\partial R}{\partial t}-\frac{\partial L}{\partial t}=-\frac{\partial uR}{\partial x}-\frac{\partial uL}{\partial x}+fL-fR\Rightarrow\frac{\partial\left(R-L\right)}{\partial t}=-\frac{\partial u\left(R+L\right)}{\partial x}-f\left(R-L\right),$$

$$\Rightarrow\frac{\partial}{\partial t}\left(\frac{J}{u}\right)=-\frac{\partial u\rho}{\partial x}-f\frac{J}{u}.$$

Multiplying by velocity and differentiating with respect to *x* yields:

$$\frac{\partial}{\partial x}\left(u\frac{\partial}{\partial t}\left(\frac{J}{u}\right)\right)=-\frac{\partial}{\partial x}\left(u\frac{\partial u\rho}{\partial x}\right)-\frac{\partial}{\partial x}\left(fJ\right).$$

Assuming *u* to be a function of *x* and not a function of time,

$$\frac{\partial}{\partial x}\left(\frac{\partial J}{\partial t}\right)=-\frac{\partial}{\partial x}\left(u\frac{\partial u\rho}{\partial x}\right)-\frac{\partial}{\partial x}\left(fJ\right).$$

For the left-hand side, the order of differentiation is interchangeable:

$$\frac{\partial}{\partial t}\left(\frac{\partial J}{\partial x}\right)=-\frac{\partial}{\partial x}\left(u\frac{\partial u\rho}{\partial x}\right)-\frac{\partial}{\partial x}\left(fJ\right).$$

Recall that, from ,

$$\frac{\partial J}{\partial x}=-\frac{\partial\rho}{\partial t},$$

so that the conservation equation becomes

$$\frac{\partial^{2}\rho}{\partial t^{2}}=\frac{\partial}{\partial x}\left(u\frac{\partial u\rho}{\partial x}\right)+\frac{\partial}{\partial x}\left(fJ\right).$$

For diffusive processes for which short-time behavior is of little interest, the second derivative in time can be considered negligible. The resulting equation,

$$\frac{\partial}{\partial x}\left(u\frac{\partial u\rho}{\partial x}\right)+\frac{\partial}{\partial x}\left(fJ\right)=0,$$

is integrated to yield:

$$J=-\frac{u}{f}\frac{\partial u\rho}{\partial x}.$$

There are 5 variations of this equation worth discussing.

**Case 1:** *u* and *f* are both uniform in space. In this case the flux equation becomes:

$$J=-\frac{u^{2}}{f}\frac{\partial\rho}{\partial x}.$$

Letting *u*^2^/*f* be the effective diffusivity *D_eff_* of the rods, we recognize as the traditional Fick’s law of diffusion:

$$J=-D_{eff}\frac{\partial\rho}{\partial x},$$

where *D_eff_* is uniform in space.

**Case 2:** *u* is uniform and *f* varies in space. In this case becomes:

$$J=-\frac{u^{2}}{f\left(x\right)}\frac{\partial\rho}{\partial x}=-D_{eff}\left(x\right)\frac{\partial\rho}{\partial x}.$$

In this case, the flux equation is Fick’s law of diffusion with variable diffusivity.

**Case 3:** The ratio *u*(*x*)/*f*(*x*) is constant. In this case, the flux equation can be expanded using the product rule:

$$J=-\frac{uu\left(x\right)}{f}\frac{\partial\rho}{\partial x}-\rho\frac{\partial}{\partial x}\left(\frac{uu\left(x\right)}{f}\right)=-D_{eff}\frac{\partial\rho}{\partial x}-\rho\frac{\partial D_{eff}}{\partial x}=\frac{\partial}{\partial x}\left(\rho D_{eff}\right)$$

Here the flux equation is one form of the Fokker-Planck law of diffusive flux.

**Case 4:** *u* varies with *x*, while *f* is a constant. In this case, the flux equation can be written as:

$$J=-\frac{u^{2}\left(x\right)}{f}\frac{\partial\rho}{\partial x}-u\left(x\right)\rho\frac{\partial}{\partial x}\left(\frac{u\left(x\right)}{f}\right).$$

From the product rule, the second term on the right-hand side of can be written

$$u\left(x\right)\rho\frac{\partial}{\partial x}\left(\frac{u\left(x\right)}{f}\right)=\frac{\rho}{2}\frac{\partial}{\partial x}\left(\frac{u^{2}\left(x\right)}{f}\right)$$

Again letting *u*^2^/*f* be the effective diffusivity, becomes

$$J=-D_{eff}\frac{\partial\rho}{\partial x}-\frac{\rho}{2}\frac{ \partial D_{eff}}{\partial x}.$$

The previous cases are described in detail by Schnitzer [6, p. 199].

**Case 5:** *u* and *f* vary independently with *x*. In the most general case, both speed and turning frequency will vary with position. In this case, the flux equation is:

$$J=-\frac{u^{2}\left(x\right)}{f\left(x\right)}\frac{\partial\rho}{\partial x}-\frac{u\left(x\right)}{f\left(x\right)}\rho\frac{\partial u\left(x\right)}{\partial x}.$$

Again, from the product rule:

$$\rho\frac{\partial}{\partial x}\left(\frac{u^{2}}{f}\right)=\rho u^{2}\frac{\partial}{\partial x}\left(\frac{1}{f}\right)+\frac{\rho}{f}\frac{\partial}{\partial x}\left(u^{2}\right)=\rho u^{2}\frac{\partial}{\partial x}\left(\frac{1}{f}\right)+\frac{2\rho u}{f}\frac{\partial u}{\partial x},$$

such that

$$\frac{\rho u}{f}\frac{\partial u}{\partial x}=\frac{\rho}{2}\frac{\partial}{\partial x}\left(\frac{u^{2}}{f}\right)-\frac{\rho u^{2}}{2}\frac{\partial}{\partial x}\left(\frac{1}{f}\right).$$

As a result, the flux equation becomes:

$$J=-D_{eff}\frac{\partial\rho}{\partial x}-\frac{\rho}{2}\frac{\partial D_{eff}}{\partial x}+\frac{\rho u^{2}}{2}\frac{\partial}{\partial x}\left(\frac{1}{f}\right).$$

In this case, the flux equation does not reduce to a form of the Fokker-Planck law, and information about turning frequency is necessary to determine the flux.

The diffusive flux equations in cases 2, 3 and 4, equations , , and , can be generalized as follows:

$$J=-D_{eff}\frac{\partial\rho}{\partial x}-\alpha\rho\frac{\partial D_{eff}}{\partial x}.$$

This flux equation is referred to as the modified **Fokker-Planck law of diffusive flux**, where *α* is the Ito-Stratonovich coefficient. This is the equation used for the PDE analysis in this work. In cases 2, 3, and 4, *α* = 0, 1, and 0.5, respectively. To use this equation, a value must be determined for *α*. This value was selected by considering the underlying physics of the rods, and by comparison with the Brownian Dynamics simulations. In previous work, we observed a quasi-linear relationship between the translational speed of PtAu rods and H_2_O_2_ concentration [7], [8] However, researchers have had little success in measuring a klinokinetic response for these particles. Therefore, we program the Brownian Dynamics simulation to assume a linear relationship between speed and fuel concentration, and no variation in rotational diffusivity. Recall from the above discussion, that this situation corresponds to case 4, where *α* = 0.5. To compare the PDE model to the BD simulation, the BD simulation was first run with a linear spatial gradient in speed (simulating a linear gradient in H_2_O_2_ and a linear relationship between the rod speed and the H_2_O_2_ concentration). The effective diffusivity of the simulated rods was calculated as a function of position; the results are shown as red circles in Figure SI1. The input into the PDE model is the blue fit curve shown in Figure SI1. The red line shows the BD simulations. The blue line shows the PDE (α = 0.5) with the gradient in diffusivity determined from the BD as shown in blue. Specifically, there is some variation in rotational diffusivity that is imposed by the reflective wall boundary condition which results in a reduction in the effective diffusivity near *x* = 0. This solution is not appropriate because when alpha is 0.5 then *f* should be constant, but we do not impose this. If we did, the effective diffusivity would appear quadratic and increase sharply at *x* = 0 (the no-boundary effective diffusivity is depicted by the green curve in Figure SI1).

Figure SI1: Effective diffusivity as a function of position across the width of the channel measured from a BD simulation (red circles), fit as the input for the PDE model (blue), and a linear gradient in effective diffusivity going from the maximum to the minimum values obtained in the BD simulation (black). The importance of edge effects is elucidated by the green curve, which shows what the diffusivity distribution would be in the absence of boundaries.

To complete the governing equation for concentration as a function of time and space, according to Fick’s second law, the time rate of change of the concentration is equal to the spatial gradient in flux. In this case, in the absence of advection,

$$\frac{\partial\rho}{\partial t}=\nabla\cdot\left(D_{eff}\nabla\rho+\alpha\rho\nabla D_{eff}\right),$$

Expanding and applying the product rule,

$$\frac{\partial\rho}{\partial t}=D_{eff}\nabla^{2}\rho+\left(1+\alpha\right)\nabla D_{eff}\cdot\nabla\rho+\alpha\rho\nabla^{2}D_{eff}$$

We use this equation for modeling the chemokinetic response of rods that lack a directional sensing component. This requires knowledge of the kinetic response of the nanorods as a function of chemical concentration, which is obtained experimentally, and knowledge of the concentration as a function of position. Furthermore, is valid for biological or synthetic nanorods that undergo pure chemokinesis (under the conditions prescribed above, where the modified Fokker-Planck law of diffusive flux is valid).

To solve the governing PDE for transport of the rods , we use a 2^nd^ order center difference method in space and a first order backward Euler method in time. This method is inherently stable and does not require any CFL type step size condition to be met. This approach requires both an initial condition and boundary conditions. The equation is solved in 1D because the chemical concentration gradient exists only in 1 dimension (*x*) in the experiments (across the width of the channel). For the initial condition we use a uniform distribution of the nanorods across the width of the channel. For the boundary conditions we establish a no-flux condition. From the Fokker-Planck equation , condition means that, at the walls,

$$D_{eff}\frac{\partial\rho}{\partial x}=-\alpha\rho\frac{\partial D_{eff}}{\partial x}.$$

In 1D, the governing PDE becomes

$$\frac{\partial\rho}{\partial t}=D_{eff}\frac{\partial^{2}\rho}{\partial x^{2}}+\left(1+\alpha\right)\frac{\partial D_{eff}}{\partial x}\frac{\partial\rho}{\partial x}+\alpha\rho\frac{\partial D_{eff}^{2}}{\partial x^{2}}.$$

The experiments used in this research involve static chemical concentration gradients. As a result, the effective diffusivity of the rods, which is dependent on the local chemical concentration gradient, does not vary with time. Therefore, the values of the 1^st^ and 2^nd^ spatial derivatives of the effective diffusivity at each position can be determined from the values of the effective diffusivity at each location. The effective diffusivity as a function of position across the channel width is provided as an input in the code and for comparisons it is obtained from the experiments. The discretized form of this equation is

$$\frac{\rho_{i,j}-\rho_{i,j-1}}{\Delta t}=D_{i}\frac{\rho_{i+1,j}-2\rho_{i,j}+\rho_{i-1,j}}{\Delta x^{2}}+\left(1+\alpha\right)D_{xi}\cdot\frac{\rho_{i+1,j}-\rho_{i-1,j}}{2\Delta x}+\alpha D_{xxi}\rho_{i,j}.$$

where *i* and *j* are indices in space and time respectively, the spatially-dependent effective diffusivity *D_i_* is an input into the code, and the first and second spatial derivatives *D_xi_* and *D_xxi_* are determined at the beginning of the code using 2^nd^-order centered difference methods and the input *D_i_*:

$$D_{xi}=\frac{D_{i+1}-D_{i-1}}{2\Delta x},$$

And

$$D_{xxi}=\frac{D_{i+1}-2D_{i}+D_{i-1}}{\Delta x^{2}}.$$

At the boundaries, the *D_x 1_* = *D_x 2_*, *D_x N_* = *D_x N-1_*, and *D_xx 1_* = *D_xx 2_*, *D_xx N_* = *D_xx N-1_*. At each time step, the number density *ρ* is determined using the above discretization and the Thomas algorithm [9]. The system of equations is solved numerically in MATLAB.

**Estimation of Effective Diffusivity as a function of Position in Experiments**

As discussed in the main text, the effective diffusivity data (plotted as a function of position in Figure 2g) is obtained from the experiments by analyzing the trajectories of the rods at various *x-*locations. To calculate the individual data points in Figure 2g, we used an in-house MATLAB code to calculate the average MSD of approximately 25 rods at each of 20 *x*-locations and estimate the effective diffusivity as the slope of the linear portion of the plot of MSD vs. time. Each rod is tracked for 50 seconds at 2 frames per second, yielding 100 total frames.

To obtain robust statistical estimates of the effective diffusivity, the MSD data is oversampled. Oversampling is a common procedure in particle tracking algorithms to strengthen the statistical estimates of MSD. For every single rod that is tracked for 100 frames, there are 81 “virtual rods” that can each be tracked for 20 frames (1 through 20, 2 through 21, …, 81 through 100). The effective diffusivity data at each point is determined by ensemble averaging the latter 10 time steps for each rod and each virtual rod. Considering that in a typical experiment there are about 500 rods in the field of view, oversampling amplifies the available data set from 500 to 40,500 rods. The effective diffusivities, computed using this approach, are shown in Figure 2g, and the linear least-squares fit to this data is the input to the spatially-dependent coefficient D_eff_ in the PDE model.

**Estimating Dimensionless Diffusivity Gradient in Brownian Dynamics Simulations**

In the Brownian Dynamics simulations, the gradient in effective diffusivity was imposed by varying the minimum and maximum translational speeds of the rods in the system. The dimensionless diffusivity gradient (abscissa in Figure 4b in the main text) is defined as

$$\frac{dD^{*}}{dx^{*}}\frac{1}{D_{min}^{*}}=\frac{d\left(D_{eff}/D_{B}\right)}{d\left(x/w\right)}\frac{1}{D_{eff,min}/D_{B}}=\frac{dD_{eff}}{dx}\frac{w}{D_{eff,min}},$$

where *w* is the channel width, *D_B_* = 1 μm^2^/s is the rods’ Brownian diffusivity, and the effective diffusivity is related to the self-propelled speed *U*, which varies with position *x*, by [4]

$$D_{eff}\left(x\right)=D_{B}+\frac{\left[U\left(x\right)\right]^{2}}{4D_{rot}}.$$

Here *D_rot_* is the rotational diffusivity of the rods, which is assumed independent of space (we are ignoring klinokinesis in this work). *D_rot_* is estimated based on the ensemble-averaged rotational mean-squared angular displacement of the rods, ensemble averaged over all the rods in the simulation.


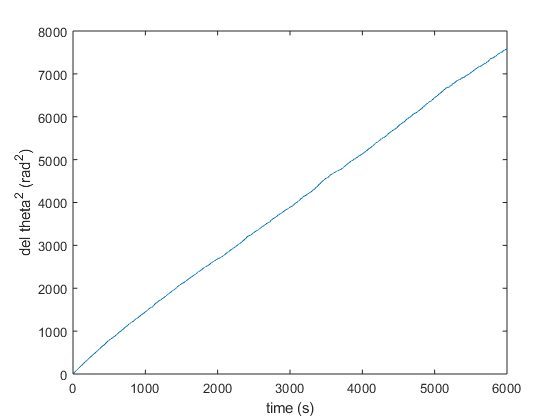


Figure SI2: Mean-squared angular displacement (ensemble-averaged over a population of 10,000 rods) vs. time for a Brownian Dynamics simulation case in which *U_min_* = 1 µm/s, *U_max_* = 20 µm/s, resulting in a steady-state CI of approximately 6.3. The value of *D_rot_* is obtained from a linear least-squares fit to this data, which clearly is very nearly linear. The value of D_rot_ for this case is 1.2487 rad^2^/s.

Figure SI2 shows the variation of Δ*θ*^2^ with time for a typical simulation run. The simulation-determined value of *D_rot_* is determined uniquely for each simulation run from a linear least-squares fit to this data, and is typically on the order of 1.1-2.0 rad^2^/s for most cases, in good agreement with the value of 1.38 rad^2^/s predicted for spheroids with the dimensions considered here by Rose et al. [10] (see equation 37 in that work). Thus, the minimum effective diffusivity in the system is

$$D_{eff,min}=D_{B}+\frac{U_{min}^{2}}{4D_{rot}}.$$

To obtain the diffusivity gradient, we need to differentiate equation with respect to *x*. Since the diffusivity depends on *U*, and since *U* also depends on *x*, we must apply the chain rule to compute the derivative:

$$\frac{dD_{eff}}{dx}=\frac{d}{dx}\left\{D_{B}+\frac{\left[U\left(x\right)\right]^{2}}{4D_{rot}}\right\}=\frac{U\left(x\right)}{2D_{rot}}\frac{dU}{dx}$$

This effective diffusivity varies roughly linearly with *x*, as shown in Figure SI1. However, as defined above, the effective diffusivity gradient varies slightly with *x*. To settle on a single value to facilitate visualization of the data in Figure 4b (main text), here we substitute the average value

$$U_{avg}=\frac{U_{\max}+U_{\min}}{2}$$

in place of *U*(*x*) in . Assuming a linear variation of velocity with *x*, the dimensionless diffusivity gradient becomes

$$\frac{dD_{eff}}{dx}\frac{w}{D_{eff,min}}\approx\frac{U_{avg}}{2D_{rot}}\left(\frac{U_{max}-U_{min}}{w}\right)\frac{w}{D_{eff,min}}$$

Simplifying, the abscissa in Figure 4b is approximated as

$$\frac{dD^{*}}{dx^{*}}\frac{1}{D_{min}^{*}}\approx\frac{1}{4}\frac{U_{max}+U_{min}}{D_{rot}}\frac{U_{max}-U_{min}}{D_{eff,min}},$$

with *D_eff,min_* given by equation . For the simulation data in Figure 4b in the main text (black circles), the abscissa is given by and the ordinate (CI) is defined using the same methodology as for the experiments.

**Diffusiophoresis**

We considered the possibility that the particles may migrate because of diffusiophoresis due to the gradient in KCl concentration. To test the effects of diffusiophoresis alone, the KCl salt gradient was established in the absence of H_2_O_2_. The nanorods are uniformly dispersed in the center channel subject to a KCl gradient. The initial distribution of rods and the resulting distribution after 33 minutes are shown in Figure SI3. In this case the distribution remains uniform; therefore, the accumulation observed in the experiments cannot be the result of KCl diffusiophoresis.


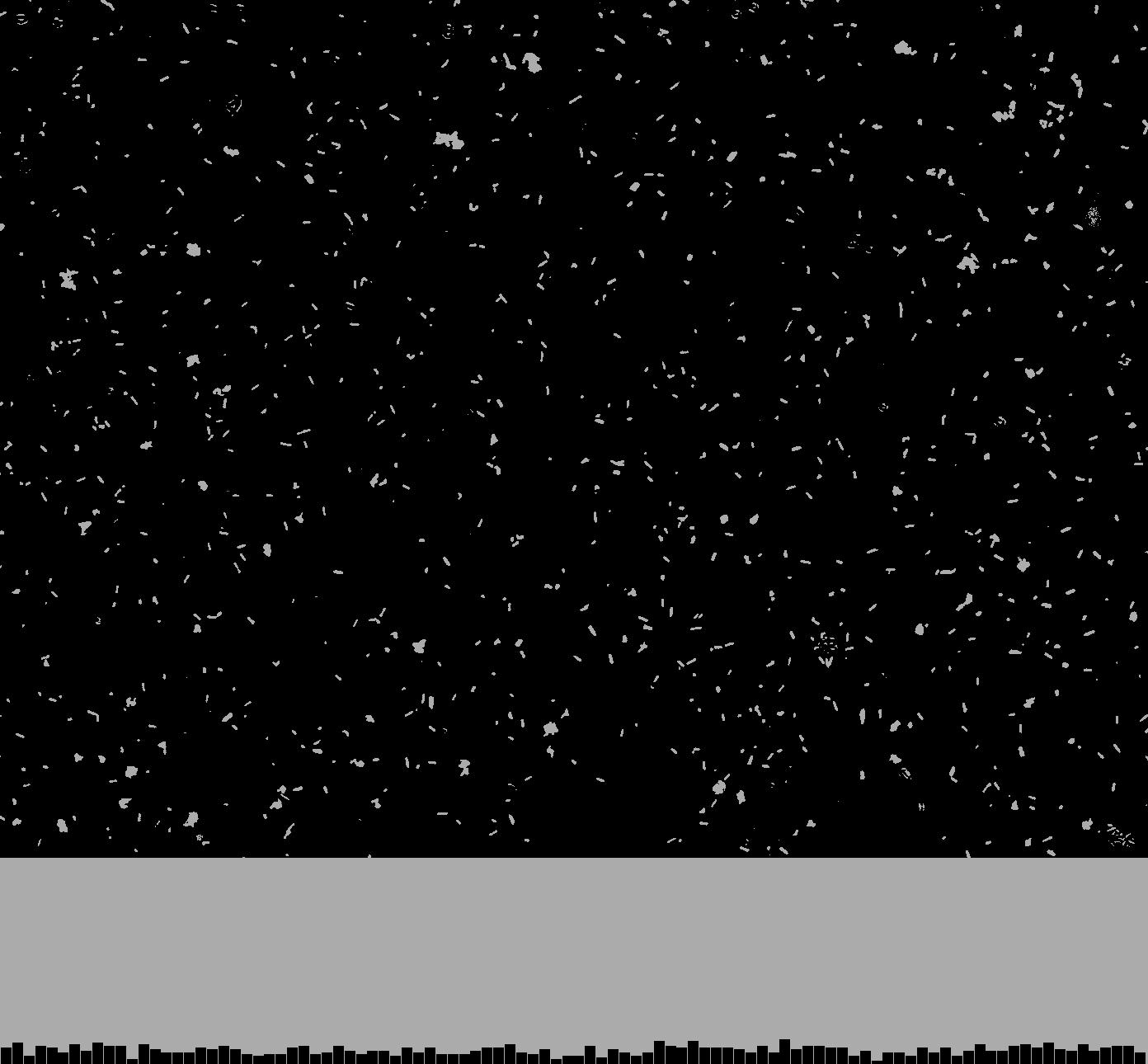

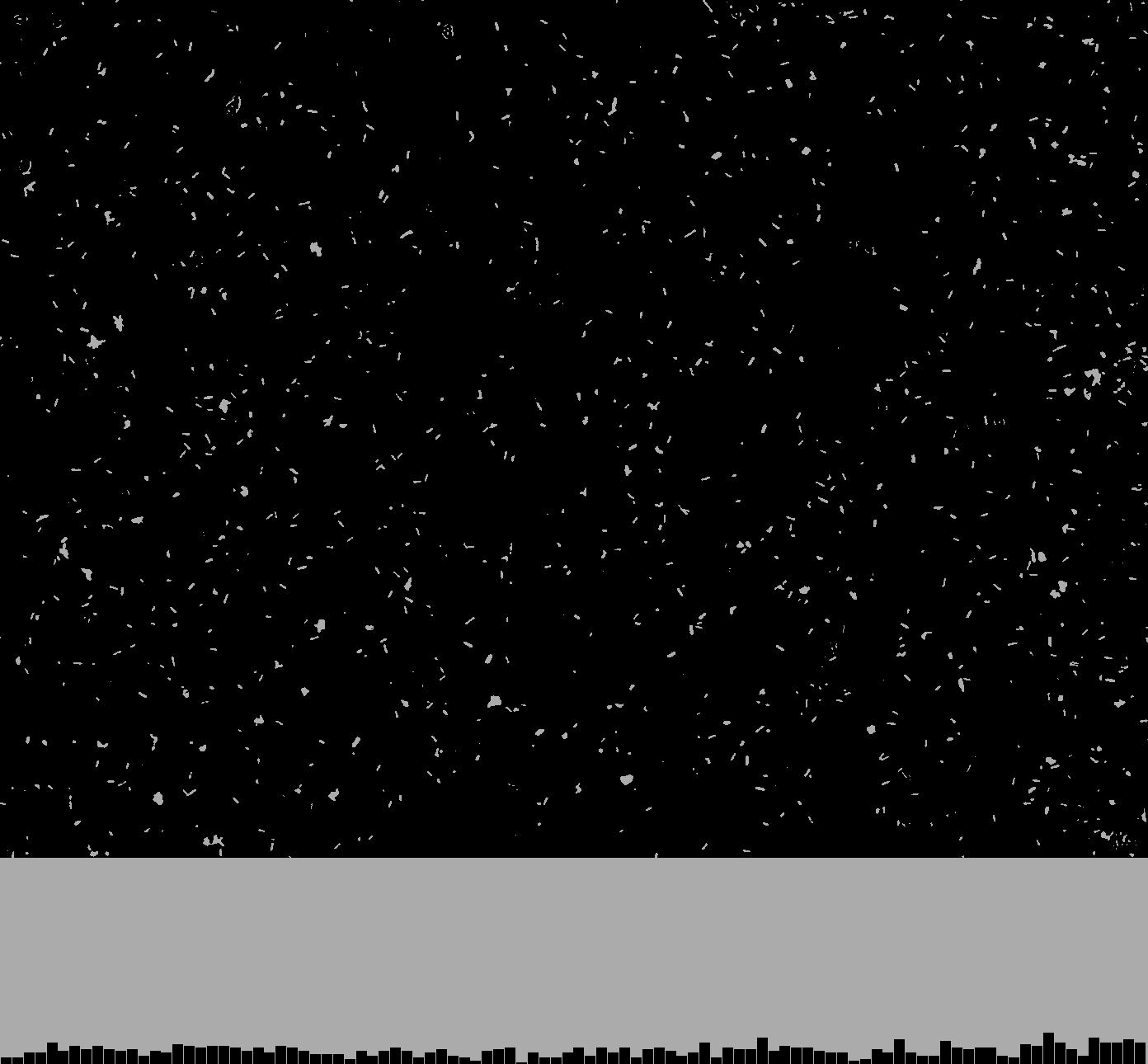


***t* = 0**

***t* = 33 min**

Figure SI3: Distribution of rods subject to a linear gradient in KCl initially (left) and after 33 minutes (right), showing no significant rod accumulation.

**Increase in Viscosity due to addition of KCl**

It is known that high concentrations of electrolyte can lead to viscosity increases. Since the rods in this work were observed to accumulate in salt-rich regions (regions of high KCl and low H_2_O_2_ concentration), one could argue that the increase in salt concentration (and the concomitant increase in viscosity) could contribute to the “trapping” of rods that is observed there. To disentangle this effect from chemokinesis, we sought to estimate the salt-induced viscosity increase in our system.

Figure SI4: Variation of viscosity of aqueous KCl solutions with KCl concentration, as tabulated in Kestin et al. 1981. The concentrations encountered in the present work cause a negligible increase in viscosity compared to water alone.

Grimes, Kestin et al. conducted a systematic investigation of the effect of adding potassium chloride (KCl) salt to deionized water [11], [12]. The data are depicted in Figure SI4. In our experiments, the maximum KCl concentration in the system considered was 0.1 mM, which was the concentration in the KCl reservoir channels (see Figure 2a in the main text). Since this reservoir channel was physically separated from the main channel by the nitrocellulose membrane, 0.1 mM is an overestimate of the maximum KCl concentration encountered by the rods. In Figure S7, the red triangle indicates the viscosity of 0.1 mM KCl in water and shows that it differs only slightly from the viscosity of pure water (the *y*-intercept in Figure SI4). From this figure, the addition of KCl causes a negligible increase in the fluid’s viscosity in our experiments. Thus, the accumulation of rods in the salt-rich regions of the channel is attributable instead to the salt-induced reduction in speed in these regions [1], [2], and the associated trapping mechanism identified and discussed in the main text. From this data, we can rule out viscosity increase as a possible explanation for the accumulation of rods in salt-rich regions.

[1] W. F. Paxton, P. T. Baker, T. R. Kline, Y. Wang, T. E. Mallouk, and A. Sen, “Catalytically induced electrokinetics for motors and micropumps,” *J. Am. Chem. Soc.*, vol. 128, no. 46, pp. 14881–14888, Nov. 2006, doi: 10.1021/ja0643164.

[2] J. L. Moran and J. D. Posner, “Role of solution conductivity in reaction induced charge auto-electrophoresis,” *Phys. Fluids 1994-Present*, vol. 26, no. 4, p. 042001, Apr. 2014, doi: 10.1063/1.4869328.

[3] D. Prieve, J. Anderson, J. Ebel, and M. Lowell, “Motion of a Particle Generated by Chemical Gradients .2. Electrolytes,” *J. Fluid Mech.*, vol. 148, no. Nov, pp. 247–269, 1984.

[4] J. R. Howse, R. A. L. Jones, A. J. Ryan, T. Gough, R. Vafabakhsh, and R. Golestanian, “Self-Motile Colloidal Particles: From Directed Propulsion to Random Walk,” *Phys. Rev. Lett.*, vol. 99, no. 4, p. 048102, Jul. 2007, doi: 10.1103/PhysRevLett.99.048102.

[5] D. Saintillan, “Rheology of Active Fluids,” *Annu. Rev. Fluid Mech.*, vol. 50, no. 1, pp. 563–592, 2018, doi: 10.1146/annurev-fluid-010816-060049.

[6] M. J. Schnitzer, “Theory of continuum random walks and application to chemotaxis,” *Phys. Rev. E*, vol. 48, no. 4, pp. 2553–2568, Oct. 1993, doi: 10.1103/PhysRevE.48.2553.

[7] J. L. Moran, P. M. Wheat, and J. D. Posner, “Locomotion of electrocatalytic nanomotors due to reaction induced charge autoelectrophoresis,” *Phys. Rev. E*, vol. 81, no. 6, p. 065302, Jun. 2010, doi: 10.1103/PhysRevE.81.065302.

[8] J. L. Moran and J. D. Posner, “Electrokinetic locomotion due to reaction-induced charge auto-electrophoresis,” *J. Fluid Mech.*, vol. 680, pp. 31–66, 2011, doi: 10.1017/jfm.2011.132.

[9] N. J. Higham, *Accuracy and Stability of Numerical Algorithms*. Society for Industrial and Applied Mathematics, 2002.

[10] K. A. Rose, J. A. Meier, G. M. Dougherty, and J. G. Santiago, “Rotational electrophoresis of striped metallic microrods,” *Phys. Rev. E*, vol. 75, no. 1, p. 011503, Jan. 2007, doi: 10.1103/PhysRevE.75.011503.

[11] C. E. Grimes, J. Kestin, and H. E. Khalifa, “Viscosity of aqueous potassium chloride solutions in the temperature range 25-150. degree. C and the pressure range 0-30 MPa,” *J. Chem. Eng. Data*, vol. 24, no. 2, pp. 121–126, 1979.

[12] J. Kestin, H. E. Khalifa, and R. J. Correia, “Tables of the dynamic and kinematic viscosity of aqueous KCl solutions in the temperature range 25–150 °C and the pressure range 0.1–35 MPa,” *J. Phys. Chem. Ref. Data*, vol. 10, no. 1, pp. 57–70, Jan. 1981, doi: 10.1063/1.555640.
